# Reduction of Retinal Thickness Ipsilateral to Hippocampal Sclerosis in Epilepsy

**DOI:** 10.3389/fneur.2021.663559

**Published:** 2021-05-11

**Authors:** Weixi Xiong, Lu Lu, Qin Chen, Yingfeng Xiao, Dongmei An, Josemir W. Sander, Ming Zhang, Dong Zhou

**Affiliations:** ^1^Department of Neurology, West China Hospital of Sichuan University, Chengdu, China; ^2^Institute of Brain Science and Brain-inspired technology of West China Hospital, Sichuan University, Chengdu, China; ^3^Department of Ophthalmology, West China Hospital of Sichuan University, Chengdu, China; ^4^The National Institute for Health Research University College London Hospitals Biomedical Research Centre, University College London Institute of Neurology, London, United Kingdom; ^5^Chalfont Centre for Epilepsy, Chalfont St Peter, United Kingdom; ^6^Stichting Epilepsie Instellingen Nederland, Heemstede, Netherlands

**Keywords:** optical coherence tomography, retinal nerve fiber layer, drug resistant epilepsy, temporal lobe epilepsy, adult with epilepsy

## Abstract

**Objectives:** Reductions in the peripapillary retinal nerve fiber layer (pRNFL) have been reported in epilepsy, namely in drug-resistant people. Hippocampal sclerosis (HS) is the most frequent cause of drug-resistant epilepsy in tertiary care centers. We aimed to evaluate the likelihood and characteristic of RNFL loss in individuals with epilepsy having HS.

**Methods:** Fifty-five adults diagnosed with unilateral HS (mean age of 25 years; 42 female) by magnetic resonance imaging were included in this observational cross-sectional study, 58 age-matched individuals with epilepsy with no detectable structural brain abnormality were included as non-HS, and 55 people without neurological diseases were included as healthy controls. pRNFL of both eyes was measured by optical coherence tomography (OCT). In each individual disease related information was recorded.

**Results:** Among the 55 individuals with unilateral HS, one (1.82%) and ten (18.18%) had significant or borderline abnormal thinning of the pRNFL of the ipsilateral eye to the HS. The average pRNFL ipsilateral to the side of HS was significantly thinner than people with epilepsy non-HS (*p* = 0.013) and healthy controls (*p* = 0.000), especially in the inferior quadrants. Only age was significantly correlated with the average and inferior quadrant pRNFL thickness of the ipsilateral eye to the HS (*R* = −0.286, *p* = 0.035; *R* = −0.353, *p* = 0.008 respectively).

**Conclusion:** These preliminary findings suggest that retinal abnormalities associated with HS may have a specific pattern. Further studies need to confirm this finding and to unravel the underlying mechanism.

## Introduction

Epilepsy is a common disease affecting up to 1% of the population (1). One third of people with epilepsy have a drug resistant condition and some are candidates for surgical treatment (2, 3). Early prediction and identification of potential candidates is crucial for considering surgery and consequent early seizure remission. Peripapillary retinal nerve fiber layer (pRNFL) thickness was recently evaluated in a cohort of people with epilepsy using optical coherence tomography (OCT), a simple, objective, high-resolution technique to quantify the RNFL. This suggested that RNFL thinning could be a potential biomarker of drug resistance and brain volume in epilepsy, with RNFL thinning much more likely to occur in people with drug-resistant epilepsy than in those who are drug responders (4). RNFL reduction (reduction in thickness) has largely been assessed in neurological and psychiatric diseases, especially Alzheimer's disease (AD). The key pathological change in AD is hippocampal sclerosis (HS), characterized by neuronal volume loss, and gliosis involving mainly CA1 and the subiculum (5). HS is also a common histopathological diagnosis in surgical resections in adults with epilepsy (6). Different from what is seen in AD, HS in epilepsy is usually unilateral. The role, if any, of HS in RNFL thickness is unknown and we aimed to explore this, particularly whether there might be asymmetries in the thickness.

We hypothesized that HS in epilepsy could be associated with a unilateral abnormality.

## Methods

### Participants

Adults attending a specialist clinic between 31 December 2016 and 1 September 2018 and diagnosed with unilateral HS were invited to participate in the study. Unilateral HS was diagnosed when unilateral atrophy of hippocampus concomitant with increased signal intensity on T2-weighted images on magnetic resonance imaging (MRI, Magnetom trio Siemens 3.0T or Siemens Sonata 1.5T, Germany) were confirmed by two independent specialists. Individuals were excluded if they were under 18 years or had: previous exposure to vigabatrin; evidence of vascular dementia; a history of brain surgery; a history of alcohol abuse; psychiatric disorders or taking psychotropic drugs; metabolic diseases; bilateral HS; arterial hypertension; other neurological diseases; previous history of high myopia above −6.00 diopters, a best-corrected visual acuity (BCVA) <20/25, optic media opacity, cataract or early lens opacity; retinal detachment; early age-related macular degeneration, or other macular degeneration; or retinal vascular diseases. This study was ethically approved by the West China Hospital Medical Ethics Committee, and informed consents were obtained from all subjects or, if subjects are under 18, from a parent and/or legal guardian. All examinations were carried out in accordance with relevant guidelines and regulations.

People with epilepsy non-HS visiting the same clinic and healthy controls without history of neurologic diseases visiting West China hospital matched on age and sex to HS people were recruited. All people with epilepsy non-HS had brain MRI just before the enrollment, and they were excluded if the MRI showed identifiable structural lesions. People with epilepsy non-HS and healthy controls were further excluded if met relevant exclusion criteria.

### Clinical Data

After informed consent, clinical data including age, sex, ethnicity, handedness, epilepsy duration (from the time of diagnosis to the date of OCT assessment) and anti-seizure medication history (ASM) were recorded.

### OCT

Peripapillary RNFL (pRNFL) of both eyes was evaluated using a spectral-domain OCT scanner (Cirrus HD-OCT 5000; Carl Zeiss Meditec, USA) with the optic disc cube 200 X 200 pattern by experienced operators blinded to the MRI result. The Cirrus HD-OCT 5000 has velocity of up to 27,000 scans/s and a 5-μm axial resolution. It also has a scanning depth of 2 mm in tissue. The scanner scans an area of 6X6 mm and provides mapping of the data of average RNFL thickness in the peripapillary area as four 90° quadrants (temporal, nasal, superior and inferior) and 12 30° sectors (from −15° to +15° for sector one and so on). The average RNFL thickness, RNFL thickness in each 90° quadrant and in each 30° sector were considered normal if they fell within the ≤95th–≥5th centile of the normal distribution percentiles provided by the manufacturer's inbuilt database, as borderline if they were between the 1st and 5th centile, and as abnormally thinned if they were <1st centile (7). The RNFL thickness of people with epilepsy non-HS and healthy controls was determined as the average of right and left eyes. All scanned images were reviewed before analysis. Images with poor quality (signal strength ≤7) or interfered by motion artifact, segmentation error were rescanned. The subjects were re-seated in front of the instrument before a second scan. The scans were discarded if a second one failed.

### Statistical Analysis

Age is shown as median (interquartile range), other data are shown as means (±Standard Deviation, SD). SPSS (version 19.0) was used for analysis. One-way analysis of variance (ANOVA) for continuous variable and Chi-square test for categorical variable were used to compare demographic data and average, quadrantile, and 30° sector RNFL thickness between people with HS, people with epilepsy non-HS and healthy controls. A Bonferroni correction was used to correct for multiple comparisons. Pearson and spearman correlation analyses were used to identify the correlations between OCT value and age, disease duration, and sex. *P* < 0.05 was considered statistically significant.

### Availability of Data and Materials

Anonymized data will be shared by reasonable requests from any qualified investigator.

## Results

Fifty-five people with HS (HS group), 58 people with MRI negative epilepsy (people with epilepsy non-HS), and 55 healthy controls were enrolled and scanned. The demographics and clinical data of 168 subjects were shown in Table 1. All were of Han ethnicity and right handed. No statistic difference was noticed between three groups.

**Table 1:** Demographic and clinical features.

	**HS**	**Epilepsy non-HS**	**Healthy controls**	***P***
Age [yr, P50 (P25, P75)]	25 (21, 33)	26 (21.75, 34.25)	31 (25, 38)	0.18^a^
Sex (M/F)	32/23	32/26	25/30	0.85^a^
Duration (yr)	9.95 ± 6.99	12.82 ± 8.77	–	0.07
Drug resistant (No.)	34	30	–	0.55

*HS, hippocampal sclerosis*.

a *Intergroup comparisons corrected by Bonferrroni correction were all >0.05*.

### OCT Examination

#### Average pRNFL Thickness Between HS and Epilepsy Groups

One of 55 participants (1.82%) in people with HS group and one of 58 (1.72%) in epilepsy non-HS had abnormal overall average pRNFL thinning (≤1st centile), with 11 further individuals (20.0%) with borderline changes (≤5th–>1st centile) in HS group and one (1.72%) in non-HS. The average RNFL of the ipsilateral eye to the HS side was significantly thinner in HS group compared to non-HS (*p* = 0.013, Table 2) and healthy controls (*p* = 0.000, Table 2). In HS group, 19 eyes reported abnormal with/or borderline changes, eleven were eyes ipsilateral to the sclerotic site and eight were contralateral, and the borderline attenuation of pRNFL in the contralateral eye was mostly accompanied with the thinning of pRNFL in the ipsilateral eye except for one individual (Table 3).

**Table 2:** The RNFL thickness comparison in people with HS, epilepsy non-HS and healthy controls.

**RNFL thickness, μm**	**HS groups**		**Epilepsy non-HS**	**Healthy controls**
	**Ipsilateral eye**	**Contralateral eye**		
Average RNFL thickness across all 4 quadrants, mean ± SD	92.64 ± 10.58^*^^,^ ^*##*^	95.29 ± 10.43^##^	97.62 ± 9.17	107.96 ± 8.99
Superior quadrant	117.73 ± 16.84^#^	122.44 ± 18.01	122.49 ± 15.54	125.24 ± 14.14
Nasal quadrant	61.51 ± 10.52^*^	60.98 ± 9.84^##^	62.66 ± 10.28	72.76 ± 12.07
Inferior quadrant	119.71 ± 20.10^*^^,^ ^*##*^	123.09 ± 18.61^##^	128.88 ± 16.52	137.91 ± 21.52
Temporal quadrant	72.47 ± 12.01^##^	74.76 ± 13.90^##^	75.04 ± 10.85	83.31 ± 15.97
30° 1	117.02 ± 26.35	124.09 ± 28.72	120.88 ± 25.89	121.67 ± 23.25
30° 2	103.18 ± 24.43	110.76 ± 25.03	111.53 ± 20.50	106.84 ± 17.77
30° 3	72.85 ± 17.10^##^	74.76 ± 18.56^##^	75.45 ± 15.95	87.90 ± 19.59
30° 4	52.82 ± 11.19^##^	54.36 ± 12.34^##^	54.25 ± 10.59	62.95 ± 11.59
30° 5	58.04 ± 9.93^##^	59.13 ± 12.69^##^	59.39 ± 10.80	65.82 ± 10.54
30° 6	90.36 ± 20.76^##^	91.76 ± 24.20^##^	98.89 ± 19.17	104.91 ± 25.25
30° 7	126.40 ± 30.35^##^	127.18 ± 31.79^##^	137.72 ± 26.26	147.91 ± 29.88
30° 8	142.40 ± 18.71^##^	140.58 ± 24.17^*^^,^ ^*##*^	149.83 ± 17.03	162.22 ± 26.68
30° 9	76.75 ± 16.54^#^	83.38 ± 30.31	77.03 ± 13.95	85.65 ± 20.54
30° 10	57.02 ± 8.95^##^	61.58 ± 12.45	59.25 ± 7.86	64.56 ± 12.47
30° 11	83.60 ± 15.65^##^	85.93 ± 20.92	88.76 ± 16.74	98.91 ± 20.74
30° 12	132.82 ± 22.18^##^	130.11 ± 27.20^##^	134.41 ± 18.61	146.71 ± 22.80

*RNFL, retinal nerve fiber layer; HS, hippocampal sclerosis*.

* *P < 0.05 after Bonferroni correction compared to the average of both eyes in epilepsy non-HS group*.

## *P < 0.01*;

# *P < 0.05 after Bonferroni correction compared to the average of both eyes in healthy controls. Comparisons between the epilepsy non-HS group and healthy controls were not shown*.

**Table 3:** The abnormal/borderline thinning of average RNFL in individuals with HS.

**No**.	**HS side**	**Age(year)**	**Sex**	**Disease duration(year)**	**History**	**Drug resistant**	**OCT result of ipsilateral eye**	**OCT result of contralateral eye**
2	L	47	M	4	/	Yes	B	B
12	L	31	M	7		No	A	N
18	R	40	M	16	/	No	B	B
19	L	35	M	16	Possible FS	Yes	B	N
21	L	40	M	16	/	Yes	B	B
30	L	44	M	6	Head trauma	Yes	B	N
120	L	22	M	5	/	Yes	B	B
133	L	23	F	23	FS	Yes	B	B
147	R	20	F	14	/	Yes	N	B
148	L	25	M	3	/	Yes	B	B
153	R	33	F	29	FS	Yes	B	B
155	L	26	M	14	FS	Yes	B	N

*HS, hippocampal sclerosis; RNFL, retinal nerve fiber layer; HS, hippocampus sclerosis; No., number; A, abnormal (≤ 1st centile); B, borderline (≤ 5th–>1st centile); N, normal (>5th centile); FS, febrile seizure*.

#### Quadrant pRNFL Thickness Between HS and Epilepsy Groups

Quadrant abnormal with/or borderline were found in 34 eyes (30.91%) in epilepsy HS group and 27 (23.28%) in epilepsy non-HS respectively. Incidence of Inferior abnormality with/or borderline in both ipsilateral and contralateral was notably higher in HS group than non-HS (Table 4) and the thickness of inferior quadrant ipsilateral to HS was significantly thinner than that in non-HS (*p* = 0.033, Table 2) and healthy controls (*p* = 0.000, Table 2). The further 30° sector analyses found a significant difference from the 3rd to 12th sectors of ipsilateral eyes compared to the healthy controls but no significance was found between the HS groups and non-HS (Table 2).

**Table 4:** Quadrant RNFL thickness, considered as categorical variable according to the normal distribution percentiles provided by the manufacturer's inbuilt database in individuals with HS and epilepsy non-HS.

	**HS (No. A/B)**		**Epilepsy non-HS (No. A/B)**
	**Ipsilateral**	**Contralateral**	
Superior quadrant	3/5	3/2	5/3
Nasal quadrant	1/6	1/4	4/12
Inferior quadrant	3/11	3/3	0/3
Temporal quadrant	0/2	0/1	0/2

*RNFL, retinal nerve fibre layer; HS, hippocampus sclerosis; No., number; A, abnormal (≤ 1st centile); B, borderline (≤ 5th to >1st centile)*.

#### RNFL Thickness and Clinical Characteristics

A significant correlation was found between the average pRNFL thickness (*R* = −0.286; *p* = 0.035) and the inferior quadrant thickness (*R* = −0.353; *p* = 0.008) of the ipsilateral eye to the age. No other correlation between the average or quadrant pRNFL thickness and the age, sex, or disease duration was found after statistical analysis (data not shown).

## Discussion

Epilepsy with unilateral HS seems to be associated with a significant pRNFL reduction pattern ipsilateral to the HS site.

There is growing evidence that retinal changes may provide a directly observable “window” to identify CNS abnormalities mainly in neurodegenerative diseases such as AD (8), PD (9), multiple sclerosis (10), and other conditions such as epilepsy (4), and schizophrenia (11). The retina and brain share common embryonic origin, while the retina has distinct layers containing gray matter. RNFL may be a quantifiable biomarker of several brain disorders.

Causes of RNFL thickness changes in neurological diseases are largely unknown. Neuroinflammatory response, mitochondrial dysfunction, dopaminergic cell loss, deposition of Aβ, and pTau have been advanced as possible reasons for RNFL reduction (12, 13). A previous epilepsy study showed that 12.7% of people with epilepsy had abnormal average RNFL thinning and 17.0% borderline attenuation, which is much higher compared to our study (1.82/20.0% in epilepsy HS and 1.72/1.72% in non-HS) (4). A possible explanation for this might be the difference of the subjects, in our study, subjects had shorter disease duration (mean 9.95 in epilepsy HS and 12.82 in non-HS) and lower drug resistant rate (61.82% in epilepsy HS and 51.72% in non-HS) compared to the prior study (mean 23.3 years and 72.2% drug-resistant epilepsy). One possible explanation might be the damage to remote regions caused by focal brain lesions through trans-synaptic degeneration over time (14, 15). ASM might contribute to the RNFL thinning, including Vigabatrin and our previous study also suggested a pattern of RNFL thinning associated with valproate using (16, 17).

As most studies assess RNFL either in one eye or using the average of both, less is known about unilateral changes. In PD, several studies reported intraocular asymmetry in the retinal thickness, as well as the extent of vessel changes, with more seen in the eye contralateral to the more affected body side, supporting an asymmetry of the neurodegenerative process involving the substantia nigra and the eye on the same side (18, 19); this is similar to our finding. In our study, the eye ipsilateral to the sclerotic site was more likely to be affected, sometimes accompanied by the thinning of RNFL in the contralateral eye. It is possible that individuals with lower RNFL values have widespread abnormalities of brain organization, which may negatively affect post-surgical outcome (20). We used strict exclusion criteria to ensure that none of the participants had a significant difference between eyes due to ocular pathology. When taking into account the fact that nasal axons cross in the chiasma, it is difficult to explain that we did not identify differences between the nasal and temporal pRNFL, as some PD studies reported changes in the nasal segments of the hemi retinae corresponding to the hemisphere mostly affected (21).

It is also difficult to explain the specific abnormal pattern of severe damage to the ipsilateral inferior quadrant, which is slightly different from the previous epilepsy study of both superior and inferior quadrant (4). The patterns of RNFL damage seem to be different under different disease conditions. For example, in PD, average and temporal pRNFL thickness seemed to be most affected, while in AD, average and all quadrants were damaged, with a preferential thinning in the superior quadrant, and in MS all quadrants except nasal thickness were found to be significantly reduced (22–24); these differences may be due to different pathogenic mechanisms. The average pRNFL seems, however, to be the most sensitive indicator in all pathologies investigated so far, which in our study, also showed significant correlation with the disease itself.

Different from the previous study (4), only correlations between the average pRNFL thickness (*R* = −0.286; *p* = 0.035) and the inferior quadrant thickness (*R* = −0.353; *p* = 0.008) of the ipsilateral eye to the age were found. The previous study revealed a correlation between disease duration and the RNFL thickness instead of the age. This discrepancy could be attributed to the nature of HS. HS was widely considered as an acquired pathology with insult, mostly febrile convulsion at young age to the immature hippocampus as initial precipitating injury long before the unprovoked seizure or epilepsy, which means the development of HS started before the epilepsy and the severity of damage to hippocampus or RNFL could be independent to the disease duration.

There are several limitations to our study. Firstly, we did not have pathological confirmation of HS diagnoses. The neuroimaging diagnoses we used are the most frequently used and validated criteria in clinical settings worldwide. MRI can reliably diagnose HS but the diagnostic gold standard is histopathological confirmation (25). Secondly, hippocampal volumes were not quantified and this could play a role. Further studies in larger cohorts of people with HS pre-and post-surgery should consider these and could provide solid evidence of a causal-relationship.

## Conclusion

Our findings suggest an association between HS and a reduction in RNFL ipsilateral to the sclerotic side. Further studies are required to clarify the underlying pathophysiology of HS as this may shed some light on the association we have seen.

## Data Availability Statement

Reasonable requests from any qualified investigator is required for the sharing of anonymized data.

## Ethics Statement

The studies involving human participants were reviewed and approved by West China Hospital Medical Ethics Committee. The participants provided their written informed consent to participate in this study.

## Author Contributions

WX analyzed the data and wrote the first draft. LL, YX, and DA helped collecting the data. JS revised the article. MZ and DZ contributed to the conception and organization of the study. All authors contributed to the article and approved the submitted version.

## Conflict of Interest

The authors declare that the research was conducted in the absence of any commercial or financial relationships that could be construed as a potential conflict of interest.
